# Associations with response to Poly(ADP-ribose) Polymerase (PARP) inhibitors in patients with metastatic breast cancer

**DOI:** 10.1038/s41523-022-00405-1

**Published:** 2022-03-31

**Authors:** A. Desnoyers, M. Nadler, B. E. Wilson, S. Stajer, E. Amir

**Affiliations:** 1 Division of Medical Oncology & Hematology, Department of Medicine, Princess Margaret Cancer Centre and the University of Toronto, Toronto, ON Canada; 2 University of New South Wales, Kensington, NSW Australia

**Keywords:** Outcomes research, Breast cancer

## Abstract

PARP inhibitors (PARPi) have modest antitumor activity in patients with advanced breast cancer and mutation in *BRCA*. It is unclear whether some subgroups derive greater benefit from treatment. MEDLINE and EMBASE were searched from inception to March 2021 to identify trials of PARPi in patients with metastatic breast cancer. Objective response rate (ORR) and clinical benefit rate (CBR) to PARPi were extracted and pooled in a meta-analysis using the Mantel Haenszel random effects model. Meta-regression explored the influence of patient and tumor characteristics on ORR and CBR. For randomized trials, hazard ratio comparing PARPi to control therapy were pooled using inverse variance and random effects. Analysis included 43 studies comprising 2409 patients. Among these, 1798 (75%) patients had *BRCA* mutations and 1146 (48%) were triple negative. In 10 studies (28%; *n* = 680 patients), the PARPi was given in combination with platinum-based chemotherapy. Weighted mean ORR was 45%; 64% when combined with platinum vs 37% with PARPi monotherapy (*p* < 0.001). Previous platinum-based chemotherapy was associated with lower ORR (*p* = 0.02). Compared to standard chemotherapy, progression-free survival was improved (HR 0.64, *p* < 0.001), but there was no difference in overall survival (HR 0.87, *p* = 0.06). There were no differences in ORR or CBR between *BRCA1* and *BRCA2* mutations. PARPi are more active in combination with platinum than as monotherapy, with lower response if given as monotherapy after platinum exposure. Significant improvements in ORR translated to modest improvement in progression-free, but not overall survival. There was no association between ORR and *BRCA* mutations.

## Introduction

Over the last decade, poly-ADP ribose polymerase (PARP) inhibitors (PARPi) have emerged as a new treatment option for metastatic breast cancer (MBC)^1^. PARP is a family of enzyme involved in base excision repair of single-strand DNA breaks^2^. When PARP is inhibited, single-strand breaks persist and result in stalled replication forks and double-strand breaks^3^. There are currently 5 PARPi being investigated in clinical trials for MBC: olaparib, rucaparib, niraparib, talazoparib and veliparib. They all differ in their potency for catalytic inhibition and their ability to trap PARP^4^. Currently, only olaparib and talazoparib are approved by the US Food and Drug Administration (FDA) and the European Medicines Agency (EMA) for use in MBC with a germline breast and ovarian cancer susceptibility gene (*BRCA*) mutation.

In MBC clinical trials, PARPi have been studied in 2 settings. The first setting is as monotherapy in cancers with impaired DNA-damage repair pathway. MBC with a germline or somatic mutation in *BRCA1* or *BRCA2* presents with deficiency in homologous recombination repair of double-stranded DNA breaks. Treatment of these BRCA-mutant cancers with PARPi leads to an accumulation of DNA damage and results in cell-cycle arrest and apoptosis. This effect is called synthetic lethality^3,5,6^. The second strategy is in combination with DNA-damaging chemotherapy, such as alkylators and/or topoisomerase I inhibitors. PARPi seem to sensitize tumor cells to damage caused by cytotoxic agents and may potentiate their effects.

PARPi when given as monotherapy or in combination with chemotherapy, are emerging treatment options in the metastatic setting for patients with breast cancer. Clinical activity is modest and selecting patients most likely to benefit from treatment is challenging. Here, we present a meta-analysis of the pooled response rates of PARPi for MBC. We explored the impact of tumor and patient level variables such as receptor status, type of combination agent and BRCA status on cancer outcomes in an attempt to better understand predictors of response in MBC.

## Results

### Study selection

**Fig. 1:**
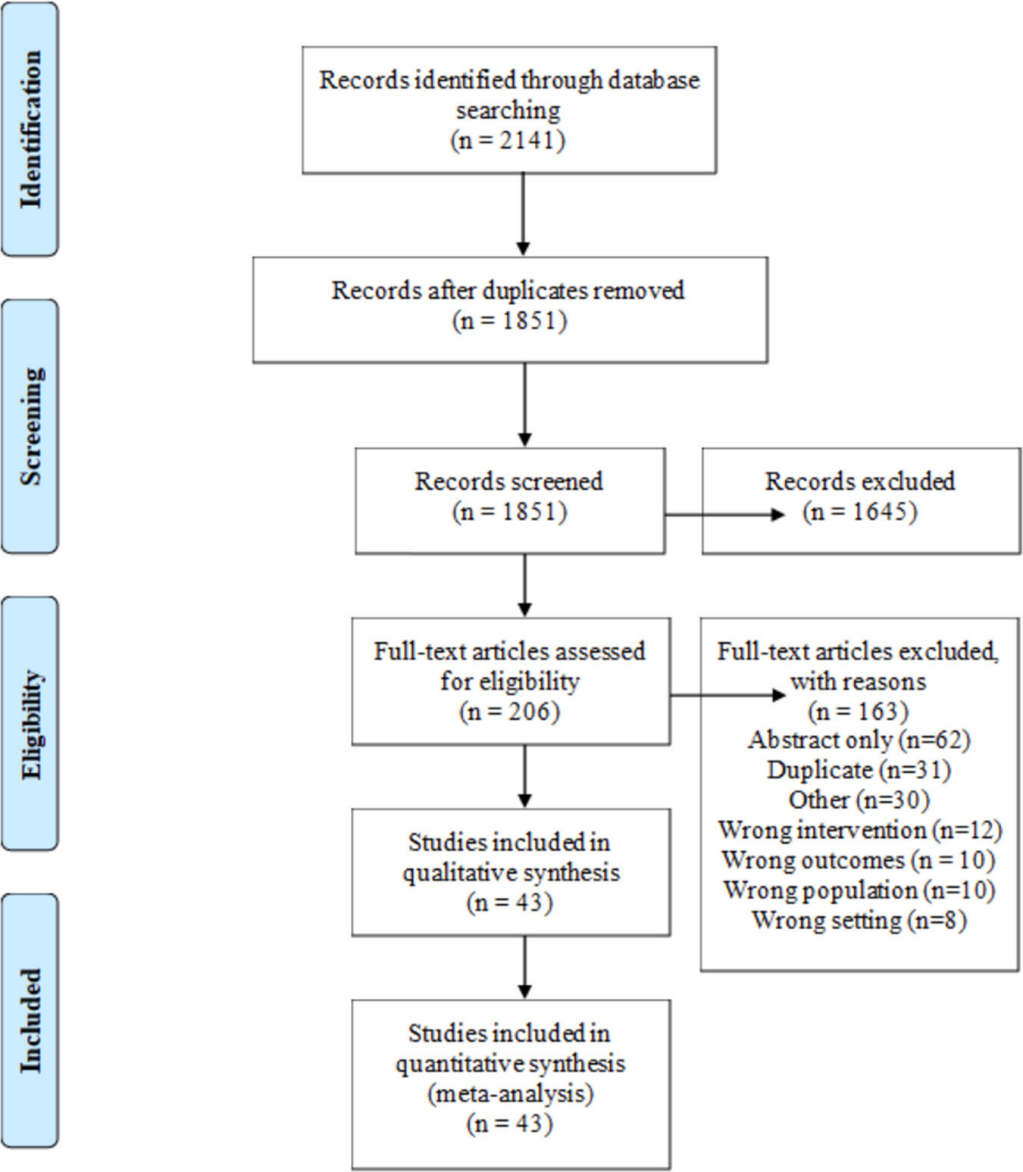
PRISMA Flow diagram.

The search identified 2141 citations of which a total of 43 studies were included in the meta-analysis (see Fig. 1 for study selection schema). Data on risk of bias of each included study are presented in SUPPLEMENTARY Table 1.

### Characteristics of the included studies

Included studies^7–49^ comprised 2409 patients. Among these, 1798 (74.6%) patients had a germline (*n* = 1783) or a somatic (*n* = 15) *BRCA* mutation. Forty-eight percent of included patients were triple-negative (TNBC; *n* = 1146). In 10 of the included studies (28%, *n* = 680 patients), the PARPi was given in combination with a platinum-based chemotherapy. Sixty-four percent (*n* = 1550) of patients were treatment-naive in the metastatic setting and 20% (*n* = 472) were previously exposed to a platinum-based chemotherapy either in the early-stage or metastatic setting (see Table 1). Five studies were randomized comparative trials whereas the remaining 38 studies were non-comparative studies. Formal assessment of publication bias was limited by a small number of comparative trials. As such funnel plots (see Supplementary Figure 1) were not informative.

**Table 1:** Characteristics of included trials (^*^Randomized trials).

Study identification number	Trial	Year of publication	Trial Phase	PARP inhibitor	Combination drug	Sample size (*n*)	Age (median)	Previous platinum, *n* (%)	Platinum refractory, *n* (%)	HR positive, *n* (%)	TNBC, *n* (%)	BRCA1, *n* (%)	BRCA2, *n* (%)	BRCA1 and/or BRCA2, *n* (%)
NCT02034916	ABRAZO	2018	2	Talazoparib	—	84	50	49 (58)	0 (0)	49 (58)	35 (42)	41 (49)	42 (50)	83 (99)
NCT01351909	Anampa	2018	1	Veliparib	Cyclophosphamide	31	52	—	—	17 (55)	14 (45)	3 (10)	5 (16)	8 (26)
NCT00535119	Appleman	2019	1	Veliparib	Carboplatin + Paclitaxel	16	—	—	—	—	—	—	—	—
NCT00782574	Balmana	2014	1	Olaparib	Cisplatin	42	—	—	—	—	—	—	—	17 (40)
NCT01123876	Berlin	2018	1	Veliparib	Folfiri	9	—	—	—	—	—	—	—	—
NCT01506609	BROCADE VCP arm	2018	2^*^	Veliparib	Carboplatin + Paclitaxel	97	44	0 (0)	0 (0)	57 (59)	40 (41)	51 (53)	44 (45)	95 (98)
NCT01506609	BROCADE VT arm	2018	2^*^	Veliparib	Temozolomide	94	46	—	—	54 (57)	38 (40)	49 (52)	43 (46)	92 (98)
NCT02163694	BROCADE3	2020	3^*^	Veliparib	Carboplatin + Paclitaxel	337	47	27 (8)	0 (0)	174 (52)	163 (48)	177 (53)	167 (50)	337 (100)
NCT01286987	De bono	2017	1	Talazoparib	—	20	—	6 (30)	0 (0)	9 (45)	9 (45)	8 (40)	11 (55)	19 (95)
NCT00819221	Del Conte	2014	1	Olaparib	Liposomal doxorubicin	13	—	—	—	—	—	—	—	—
NCT00707707	Dent	2013	1	Olaparib	Paclitaxel	19	50	—	—	0 (0)	19 (100)	—	—	—
NCT00664781	Drew	2016	2	Rucaparib	—	27	—	—	—	—	—	15 (56)	12 (44)	27 (100)
NCT01945775	EMBRACA	2020	3^*^	Talazoparib	—	287	45	46 (16)	0 (0)	157 (55)	130 (45)	133 (46)	154 (54)	287 (100)
NCT00516373	Fong	2009	1	Olaparib	—	9	—	—	—	—	—	0 (0)	3 (33)	3 (33)
NCT00679783	Gelmon	2011	2	Olaparib	—	26	47	—	—	5 (19)	21 (81)	4 (15)	6 (23)	10 (38)
NCT02401347	Gruber	2019	2	Talazoparib	—	13	—	—	0 (0)	12 (92)	1 (8)	0 (0)	0 (0)	0 (0)
NCT02498613	Hafez	2020	2	Olaparib	Cediranib	37	49	34 (92)	30 (81)	0 (0)	37 (100)	—	—	—
NCT00494234	ICEBERG 1	2010	2	Olaparib	—	54	45	14 (26)	5 (9)	20 (37)	29 (54)	33 (61)	20 (37)	54 (100)
NCT03330405	JAVELIN PARP Medley	2020	2	Talazoparib	Avelumab	22	—	—	—	3 (14)	19 (86)	—	—	—
NCT01078662	Kaufman	2015	2	Olaparib	—	62	48	42 (68)	—	32 (52)	30 (48)	37 (60)	25 (40)	62 (100)
NCT01482715	Kristeleit	2017	1	Rucaparib	—	27	—	—	—	—	—	6 (32)	5 (26)	11 (58)
NCT00810966	Kummar	2012	1	Veliparib	Cyclophosphamide	12	—	—	—	—	—	—	—	—
NCT01306032	Kummar	2016	2^*^	Veliparib	Cyclophosphamide	21	54	—	—	0 (0)	21 (100)	—	—	2 (10)
NCT01445418	Lee	2017	1	Olaparib	Carboplatin	28	51	3 (11)	—	0 (0)	28 (100)	0 (0)	0 (0)	0 (0)
NCT01237067	Lee	2016	1	Olaparib	Carboplatin	14	—	—	—	3 (21)	11 (79)	3 (21)	4 (29)	7 (50)
NCT0328684	LUCY	2020	3	Olaparib	—	252	—	81 (32)	—	131 (52)	121 (48)	136 (54)	104 (41)	252 (100)
NCT01623349	Matulonis	2017	1	Olaparib	Buparlisib	24	48	—	—	11 (46)	13 (54)	—	—	15 (63)
NCT02734004	MEDIOLA	2020	2	Olaparib	Durvalumab	34	46	13 (38)	—	16 (47)	18 (53)	16 (47)	18 (53)	34 (100)
NCT02000622	OlympiAD	2019	3^*^	Olaparib	—	205	44	60 (29)	0 (0)	103 (50)	102 (50)	117 (57)	84 (41)	205 (100)
NCT01281150	Pahuja	2015	1	Veliparib	Carboplatin + Paclitaxel	24	—	—	—	0 (0)	22 (92)	—	—	9 (38)
NCT01145430	Pothuri	2020	1	Veliparib	Pegylated liposomal doxorubicin	11	—	0 (0)	0 (0)	0 (0)	11 (100)	—	—	—
NCT00892736	Puhalla	2014	1	Veliparib	—	40	—	40 (100)	40 (100)	—	—	—	—	16 (40)
NCT01104259	Rodler	2016	1	Veliparib	Cisplatin + Vinorelbine	50	50	17 (34)	—	9 (18)	41 (82)	10 (20)	4 (8)	14 (28)
NCT00749502	Sandhu	2013	1	Niraparib	—	12	—	—	1 (8)	—	—	—	—	4 (33)
UMIN000018721	Shimomura	2019	1	Olaparib	Eribulin	32	—	—	—	0 (0)	32 (100)	5 (16)	0 (0)	5 (16)
ANZCTRN12613000924752	SOLACE	2019	1	Olaparib	Cyclophosphamide	9	46	6 (67)	—	6 (67)	3 (33)	2 (22)	4 (44)	6 (67)
NCT01149083	Somlo	2017	1	Veliparib	Carboplatin	28	45	0 (0)	0 (0)	19 (68)	8 (29)	12 (43)	15 (54)	27 (96)
NCT01149083	Somlo	2017	2	Veliparib	—	44	43	0 (0)	0 (0)	22 (50)	—	22 (50)	22 (50)	44 (100)
NCT01154426	Stoller	2017	1	Veliparib	Gemcitabine	8	—	—	—	0 (0)	—	—	—	—
NCT02158507	Stringer-Reasor	2021	1	Veliparib	Lapatinib	17	—	—	—	0 (0)	17 (100)	—	—	—
NCT03344965	TBCRC 048 cohort 1	2020	2	Olaparib	—	27	54	0 (0)	0 (0)	23 (85)	2 (7)	0 (0)	0 (0)	0 (0)
NCT03344965	TBCRC 048 cohort 2	2020	2	Olaparib	—	27	59	3 (11)	0 (0)	18 (67)	8 (30)	6 (22)	10 (37)	16 (59)
NCT02657889	TOPACIO	2019	2	Niraparib	Pembrolizumab	55	54	31 (56)	16 (29)	0 (0)	55 (100)	—	—	15 (27)
NCT01853306	Werner	2018	1	Veliparib	—	17	—	—	—	—	—	—	—	17 (100)
NCT01251874	Wesolowski	2014	1	Veliparib	Carboplatin	44	—	—	—	—	—	—	—	—
UMIN00009498	Yonemori	2019	2	Olaparib	Eribulin	24	46	—	—	0 (0)	24 (100)	2 (8)	0 (0)	2 (8)
UMIN00009498	Yonemori	2019	1	Olaparib	Eribulin	24	52	—	—	0 (0)	24 (100)	2 (8)	1 (4)	3 (13)

### Response rate

All 43 studies were included in the pooled analysis for response rate (see Table 2 for response rates reported in individual studies). Weighted mean ORR and CBR for all MBC patients were 45% (range 0–78%) and 66% (range 19–91%), respectively. Weighted mean ORR was 64% (range 19-78%) when the PARPi was combined with a platinum versus 37% (range 0–63%) when it was not (*p*<0.001). Weighted mean CBR was 84% (range 44–91%) when the PARPi was combined with a platinum versus 59% (range 19–86%) when it was not (*p*<0.001). Weighted mean ORR and CBR were 21% and 51% for niraparib, 38% and 57% for olaparib, 7% and 38% for rucaparib, 51% and 72% for talazoparib and 51% and 74% for veliparib, respectively (*p* = 0.59 and *p* = 0.56 respectively). For hormone receptor positive MBC, the pooled ORR and CBR were 57% (range 0–74%) and 91% (range 50–92%), respectively Among triple-negative MBC patients, the pooled ORR was 49% (range 0–78%) and CBR was 75% (range 33–89%) (Table 2). The differences between hormone receptor positive and triple negative tumors were not significant for ORR (*p* = 0.38), but modestly significant for CBR (*p* = 0.03). In patients with a BRCA1 and/or BRCA2 mutation, pooled ORR was 57% (range 0–100%) and pooled CBR was 73% (range 35–100%). For trials that reported response rates for BRCA1 and BRCA2 separately (*n* = 28), the pooled ORR was similar between the two groups: 43% (range 0–100%) and 46% (range 0–100%), respectively (*p* = 0.72). Similarly, the pooled CBR was 68% (range 23–100%) and 74% (range 50–100%) for BRCA 1 and BRCA2 respectively; *p* = 0.60).

**Table 2:** Overall response rate and clinical benefit rate of all included studies.

Trial	Phase	PARP inhibitor	Combination drug	ORR/CBR All patients (%/%)	ORR/CBR HR positive (%/%)	ORR/CBR TNBC (%/%)	ORR/CBR BRCA1 and/or BRCA2 (%/%)
ABRAZO, 2018	2	Talazoparib	—	28/35	29/–	26/–	28/49
Anampa, 2018	1	Veliparib	Cyclophosphamide	12/19	–/–	–/–	–/43
Appleman, 2019	1	Veliparib	Carboplatin + Paclitaxel	69/85	–/–	–/–	–/–
Balmana, 2014	1	Olaparib	Cisplatin	–/–	–/–	–/–	71/–
Berlin, 2018	1	Veliparib	Folfiri	25/−	–/–	–/–	–/–
BROCADE, 2018 VCP arm	2	Veliparib	Carboplatin + Paclitaxel	78/91	–/–	–/–	78/91
BROCADE, 2018 VT arm	2	Veliparib	Temozolomide	29/73	–/–	–/–	29/73
BROCADE3, 2020	3	Veliparib	Carboplatin + Paclitaxel	76/91	74/92	78/89	76/91
De bono, 2017	1	Talazoparib	—	50/86	–/89	–/56	47/74
Del Conte, 2014	1	Olaparib	Liposomal doxorubicin	8/–	–/–	–/–	–/–
Dent, 2013	1	Olaparib	Paclitaxel	37/68	–/–	37/68	–/–
Drew, 2016	2	Rucaparib	—	0/39	–/–	–/–	0/39
EMBRACA, 2020	3	Talazoparib	—	63/84	63/–	62/–	63/84
Fong, 2009	1	Olaparib	—	–/–	–/–	–/–	33/67
Gelmon, 2011	2	Olaparib	—	0/30	0/–	0/–	0/63
Gruber, 2019	2	Talazoparib	—	25/50	–/–	–/–	–/–
Hafez, 2020	2	Olaparib	Cediranib	–/–	–/–	–/ 81	–/–
ICEBERG 1, 2010	2	Olaparib	—	31/76	30/90	38/79	31/76
JAVELIN PARP Medley, 2020	2	Talazoparib	Avelumab	8/58	–/–	8/58	–/–
Kaufman, 2015	2	Olaparib	—	13/35	13/–	13/–	13/35
Kristeleit, 2017	1	Rucaparib	—	15/37	–/–	–/–	36/73
Kummar, 2012	1	Veliparib	Cyclophosphamide	17/33	–/–	–/–	–/–
Kummar, 2016	2	Veliparib	Cyclophosphamide	10/33	–/–	10/33	50/100
Lee, 2017	1	Olaparib	Carboplatin	19/44	–/–	19/44	–/–
Lee, 2016	1	Olaparib	Carboplatin	50/79	–/–	–/–	71/100
LUCY, 2020	3	Olaparib	—	–/49	–/–	–/–	− / 49
Matulonis, 2017	1	Olaparib	Buparlisib	28/72	–/–	–/–	33/75
MEDIOLA, 2020	2	Olaparib	Durvalumab	63/80	69/92	59/71	63/80
OlympiAD, 2019	3	Olaparib	—	60/–	65/–	55/–	60/–
Pahuja, 2015	1	Veliparib	Carboplatin + Paclitaxel	52/–	–/–	52/–	60/–
Pothuri, 2020	1	Veliparib	Pegylated liposomal doxorubicin	0/36	–/–	0/36	–/–
Puhalla, 2014	1	Veliparib	—	14/46	–/–	–/–	29/57
Rodler, 2016	1	Veliparib	Cisplatin + Vinorelbine	35/79	–/–	–/–	57/93
Sandhu, 2013	1	Niraparib	—	20/60	–/–	–/–	50/75
Shimomura, 2019	1	Olaparib	Eribulin	35/79	–/–	35/76	40/100
SOLACE, 2019	1	Olaparib	Cyclophosphamide	0/56	0/50	0/67	0/50
Somlo, 2017	1	Veliparib	Carboplatin	56/59	53/–	63/−	58/73
Somlo, 2017	2	Veliparib	—	25/43	36/–	–/–	25/43
Stoller, 2017	1	Veliparib	Gemcitabine	0/57	–/–	–/–	–/–
Stringer-Reasor, 2021	1	Veliparib	Lapatinib	24/35	–/–	24/35	–/–
TBCRC 048, 2020 Cohort 1	2	Olaparib	—	33/50	36/−	0/–	–/–
TBCRC 048, 2020 Cohort 2	2	Olaparib	—	31/48	–/–	–/–	–/–
TOPACIO, 2019	2	Niraparib	Pembrolizumab	21/49	–/–	21/49	47/80
Werner, 2018	1	Veliparib	—	25/–	–/–	–/–	25/–
Wesolowski, 2014	1	Veliparib	Carboplatin	19/67	–/–	–/–	–/–
Yonemori, 2019	2	Olaparib	Eribulin	38/75	–/–	38/75	100/100
Yonemori, 2019	1	Olaparib	Eribulin	17/75	–/–	17/75	67/100

### Meta-regression analysis

Univariable and multivariable meta-regression results are shown in the Table 3. When adjusted for covariates, having received previous platinum chemotherapy was negatively associated with ORR to PARPi among all MBC (*p* = 0.02). Similarly, there was a statistically significant negative association between ORR and platinum refractory disease (*p* = 0.03). There was a statistically significant positive association between ORR to PARPi and the proportion of patients with TNBC in individual trials (*p* = 0.03). However, no association was found for the proportion of patients with hormone receptor positive MBC (*p* = 0.33). While there was an association between BRCA mutation and ORR in univariable analysis, this association was not maintained after adjustment for other variables. While combination platinum, age, and treatment for first line MBC were associated with CBR in univariate meta-regression, there were no statistically significant association with CBR when adjusting for covariates using multivariate meta-regression.

**Table 3:** Meta-regression analysis for outcomes among included studies.

	ORR			CBR		
Univariable						
Predictor variable	Adjusted Beta coefficient	95% CI	*p*-value	Adjusted Beta coefficient	95% CI	*p*-value
Sample size	0.002	0.001–0.003	0.0002	0.001	−0.0001–0.002	0.06
HR positive	0.17	−0.11–0.46	0.22	−0.03	−0.31–0.24	0.81
TNBC	−0.20	−0.46–0.06	0.13	−0.007	−0.26–0.25	0.96
BRCA1 and/or BRCA2	0.21	0.02–0.39	0.03	0.12	−0.07–0.31	0.21
Combined with platinum	0.27	0.13–0.41	0.0004	0.20	0.05–0.34	0.01
Previous platinum	−0.35	−0.73–0.04	0.08	−0.08	−0.39–0.23	0.59
Platinum refractory	−0.32	−0.80–0.16	0.17	−0.02	−0.38–0.35	0.92
First line MBC	0.21	0.05–0.37	0.01	0.14	−0.02–0.29	0.08
Age	−0.02	−0.04–0.0001	0.05	−0.02	−0.04–−0.001	0.04
PARPI						
Niraparib	−0.11	—	0.51	−0.02	—	0.89
Olaparib	−0.03	—	0.74	0.06	—	0.45
Rucaparib	−0.24	—	0.15	−0.18	—	0.23
Talazoparib	0.03	—	0.76	0.06	—	0.57
Veliparib	REF	—	—	REF	—	—
**Multivariable**						
Age	−0.02	−0.03–−0.001	0.02	−0.02	−0.10–0.06	0.17
HR positive	0.68	−1.66–3.03	0.33	−3.25	−20.62–14.11	0.25
TNBC	1.93	0.35–3.51	0.03	−0.48	−12.03–11.07	0.69
BRCA1 and/or BRCA2	−0.30	−0.62–0.02	0.05	−0.55	−3.12–2.00	0.22
Combined with platinum	0.09	−0.04–0.25	0.09	−0.02	−0.98–0.95	0.84
Previous platinum	−0.52	−0.84–−0.20	0.02	−0.70	−3.06–1.66	0.17
Platinum refractory	−3.23	−5.84–−0.64	0.03	−6.16	−27.17–14.84	0.17

### Progression-free and overall survival

**Fig. 2:**
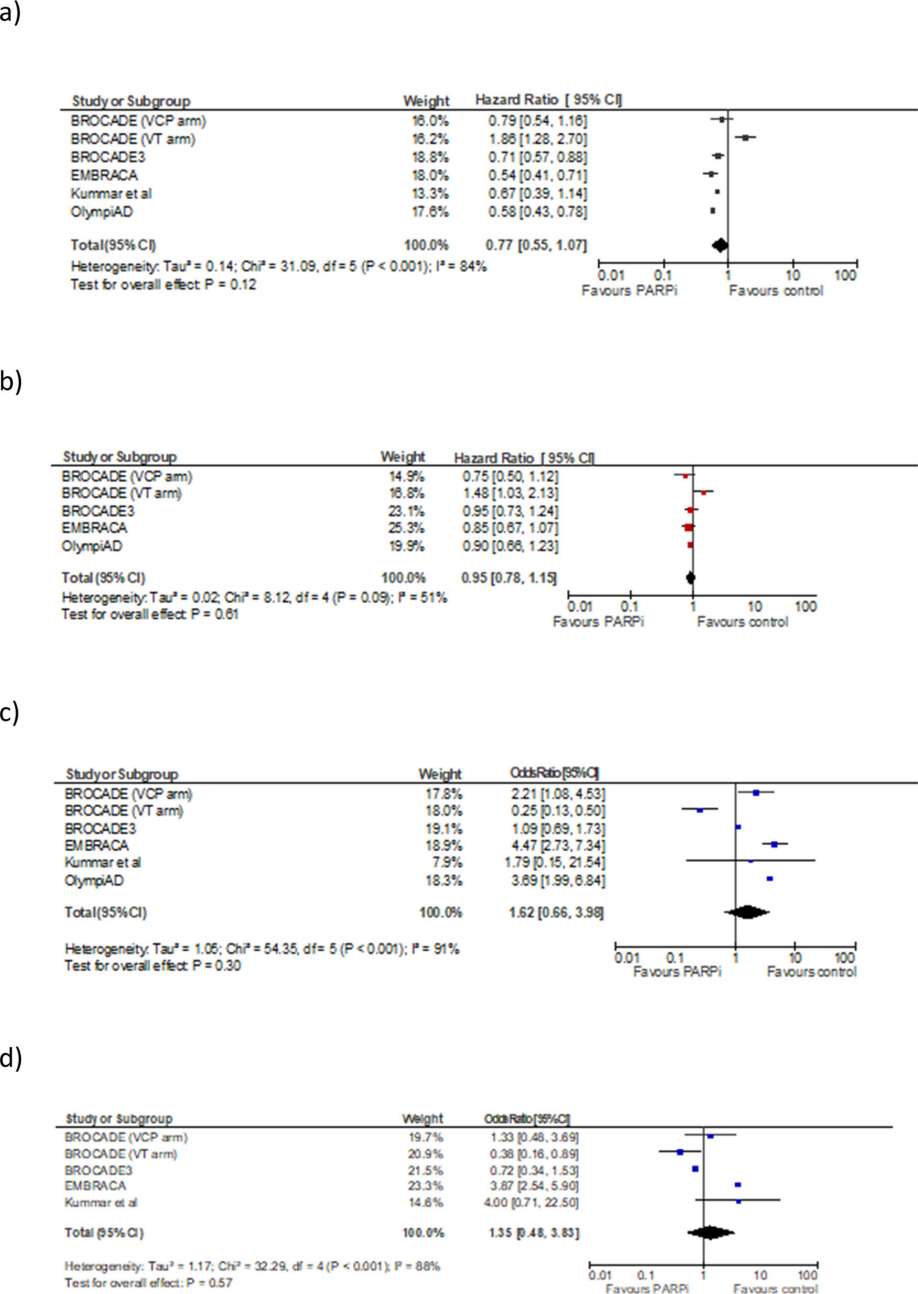
Forest Plots for randomized trials comparing PARPi to control. **a** PFS, **b** OS, **c** ORR, **d** CBR.

For one study^34^, the HR was not reported explicitly, and was estimated from survival curves using the Parmar toolkit^50^. Meta-analysis comprised 5 RCTs^12,13,26,34,37^ (1574 patients) (see Fig. 2). In the pooled analyses, the use of a PARPi versus chemotherapy in MBC patients was associated non-significantly with improved PFS (HR 0.77, 0.55–1.07, *p* = 0.12, Fig. 2a). There was significant heterogeneity (Cochran’s Q *p*<0.001, I^2^ 84%). OS was not different between both treatment arms (HR 0.95, 0.78–1.15, *p* = 0.61 Fig. 2b). Compared to chemotherapy alone, patients randomized to PARPi had non-significantly higher odds of experiencing ORR (OR 1.62, 0.66-3.98, *p* = 0.30, Fig. 2c) as well as CBR (OR 1.35, 0.48–3.83, *p* = 0.57, Fig. 2d). There was statistically significant heterogeneity for ORR (Cochran’s Q *P*<0.001, I^2^ = 91%) and CBR (Cochran’s Q *P*<0.001, I^2^ = 88%) pooled results.

Of note, the veliparib and temozolomide (VT) arm of the BROCADE trial^13^ was a source for heterogeneity. As such, data for this treatment were excluded in a sensitivity analysis. Results showed statistically significant PFS and non-significant heterogeneity (HR 0.64, 95% CI 0.56–0.75, *p*<0.001; Cochran’s Q *p* = 0.43, I^2^ 0%). For OS, data approached, but did not meet statistical significance and again heterogeneity was attenuated (HR 0.87, 95% CI 0.76–1.01, *p* = 0.06; Cochran’s Q *p* = 0.79, I^2^ 0%).

## Discussion

In unselected MBC, our study shows that PARPi are associated with a pooled ORR of 45% and a CBR of 66%. This is slightly better than the reported ORR of 35-40% with first-line single agent chemotherapy such as anthracyclines^51^ or taxanes^52^, 30% with carboplatin for BRCA mutated TNBC^53^ and significantly better than the 13% with eribulin in the third-line setting^54^. We also found a higher ORR (64%) and CBR (84%) when PARPi were combined with platinum-based chemotherapy versus when it was not (37% and 59%, respectively). This is concordant with the principles of synthetic lethality. Of note, meta-regression confirmed a significant association between previous platinum exposure and platinum refractory disease with lower response rate. This is an important observation especially as platinum resistance was an exclusion criteria for most of the PARPi RCTs performed in metastatic disease. Of note, this observation is concordant with the adjuvant Olaparib randomized phase III trial^55^, where the improvement in invasive disease-free survival was substantially lower among patients with prior exposure to platinum-based chemotherapy (*n* = 486, HR 0.77, 95% CI 0.49–1.21) compared to those without prior exposure to a platinum (*n* = 1350, HR 0.52, 95% CI 0.39–0.69).

Veliparib and talazoparib had the numerically highest response rates (ORR 51% and 51%, CBR 72% and 74%, respectively) and rucaparib the lowest (ORR 7% and CBR 38%), although these differences were not statistically significant. Response rates in patients with a *BRCA1* and/or *BRCA2* mutation were similar (ORR 57% vs 45% and CBR 73% vs 66%, respectively) and we found no association between mutation status and response rate in meta-regression analysis. These results might reflect the fact that vast majority of included patients had a *BRCA* mutation.

Of note, for the five largest trials included in this meta-analysis, pooled data did not demonstrate statistically significant PFS and/or OS improvement in unselected patients. The PFS result remained non-significant even after a sensitivity analysis was performed, excluding the one trial performed in non-BRCA mutated patients^34^. However, statistically significant PFS improvement was observed if trials were restricted to those with standard chemotherapy (i.e. excluding one study using temozolomide as the chemotherapy backbone). There was no improvement in OS in any analysis. This likely reflects the relatively modest incremental benefits of PARPi in MBC.

To our knowledge this is the largest pooled analysis of PARPi and outcomes in unselected MBC. Poggio et al^56^. reported on a pooled analysis of only two RCTs and while results were similar to those reported here, the current analysis provides greater precision for the estimates of the impact of PARPi in MBC while also supplementing the analysis with data from non-comparative, earlier phase trials. Another analysis by Wang et al^57^. aimed to compare two different PARPi rather than report on a comparison of PARPi to standard of care.

This study has limitations. First, there was substantial heterogeneity in patient population and treatment exposures. Meta-analytic methods such as weighted pooling, subgroup and sensitivity analyses and meta-regression were utilized to explore these differences. As noted in the results, a few quantitative or statistically significant differences were observed including the impact of concurrent and prior platinum. Important negative findings were also identified including the lack of quantitative or statistically significant differences between different *BRCA* mutations, ER-expression and the different PARP inhibitors. Beyond these known potential sources of heterogeneity, we also explored other (and potentially unmeasured) confounders. Sensitivity analysis excluding studies which contributed to statistical heterogeneity showed similar effect sizes. While the strategy we utilized to address heterogeneity is robust and provides clinically useful and potentially actionable information, the potential for residual heterogeneity remains and this might impact the generalizability of our results. Second, we relied on summary trial data and meta-regression rather than individual patient data. This will also add uncertainty to the results. Third, while we included many early phase trials, our reliance on published data will result in the potential for publication bias. Finally, we were unable to analyze publication bias robustly as the number of comparative trials was small. Second, we relied on summary trial data and meta-regression rather than individual patient data. This will also add uncertainty to the results. Third, while we included many early phase trials, our reliance on published data will result in the potential for publication bias. Finally, we were unable to analyze publication bias robustly as the number of comparative trials was small.

In summary, PARPi have modest activity in MBC and this activity seems greater in combination with platinum than as monotherapy. There is lesser response to PARPi monotherapy in patients with prior platinum exposure, especially platinum resistance. Significant improvements in ORR translated to modest improvement in progression-free survival in trials using standard chemotherapy. However, overall survival was not improved. There was no association between ORR and type of BRCA mutation status. Further clinical trials are needed to better characterize which patient population could benefit most from these drugs.

## Methods

### Study inclusion criteria and strategy

A systematic review consistent with Preferred Reporting Items for Systematic Reviews and Meta-Analyses (PRISMA) criteria was performed^58^. We searched EMBASE and MEDLINE databases from inception to March 18, 2021. A targeted search was performed to identify clinical studies that reported response rate for PARPi in patients with metastatic breast cancer. Inclusion criteria comprised adult women with inoperable locally advanced or metastatic breast cancer treated with a PARPi in the palliative setting. Studies included in the current analysis met additional predefined eligibility criteria (see Table 4). The detailed search strategy is reported in SUPPLEMENTARY INFORMATION 1. Citations retrieved through the literature search were screened initially for inclusion based on their title and abstract. Full texts were obtained for citations that met the inclusion criteria where it was not possible to determine whether the study fulfilled inclusion criteria based on the abstract alone.

**Table 4:** Inclusion/Exclusion criteria for studies.

Criteria	Inclusion	Exclusion
Study design	RCT, non-RCT	Observational studies, case studies, case reports
Population	Adult women (≥18 years) with advanced or metastatic breast cancer	
Disease	Metastatic breast cancer, platinum-sensitive or -resistant	
Histopathological subtype	Any histological type	
Intervention	All trials investigating PARPi in patients with metastatic breast cancer FDA-approved PARPi used alone or in combination with FDA-approved chemotherapy agents, immunotherapy, or targeted therapy	Studies investigating the role of radiotherapy, hormonal therapy, or surgery Combination with radiation therapy and/or non-FDA approved cancer drugs
Comparators	Any active chemotherapy or placebo or no comparator arm	
Line of therapy	First-line therapy or above	Neoadjuvant or adjuvant, Maintenance therapy
Language	Full-text, English-language publications	
Publication time frame	Inception to March 18th 2021	

### Data abstraction

Data extraction was performed by a single researcher (AD). Outcome data were extracted from the primary publication and/or supplemental appendices for objective response rate (ORR), clinical benefit rate (CBR), progression-free survival (PFS), and overall survival (OS). For response rate, we collected the exact number of events and the total number of patients included in the analysis. For PFS and OS, we extracted the hazard ratio (HR) and respective 95% confidence interval (CI). If the HR was not reported explicitly, it was estimated from survival curves using the Parmar toolkit^50^. Additionally, we collected the generic name of PARPi, combination drug (if any), sample size and the following study level patient characteristics: median age, number of previous lines in metastatic setting, previous platinum chemotherapy, platinum sensitivity as defined by individual trials, proportion with hormone receptor positive or triple negative disease and presence of germline *BRCA1* and *BRCA2* mutations.

### Risk of bias

Individual study risk of bias were assessed using the Cochrane risk of bias tools; RoB 2.0 tool for randomized trials and ROBINS-I tool for non-randomized studies of interventions. Bias domains included the following: pre-intervention bias (selection and randomization), bias due to deviation from intended intervention, due to missing data, due to outcome measurement and bias due to selection of reported results (see SUPPLEMENTARY Table 1).

### Study objectives

The objectives of this study were to pool and compare the activity and efficacy of PARPi for MBC in the overall population. Exploratory subgroups analyses were performed to investigate if activity differed based on hormone receptor status, *BRCA* mutation status, prior exposure to platinum-based chemotherapy, platinum sensitivity and choice of PARPi.

### Statistical analysis

Data were reported descriptively as pooled proportion, mean, and range as appropriate. Analyses were weighted by sample size to compensate for the variability in sample sizes across studies. Odd ratios (OR) were calculated between subgroups: receptor status, *BRCA* mutations and prior platinum exposure. Analyses were performed using SAS Studio 3.8 (Institute Inc., Cary, NC). Meta-regression was used to explore the influence of previous chemotherapy and patient and tumor characteristics on ORR and DCR as reported in individual studies. Meta-regression comprised a linear regression weighted by individual study sample size and using a random effects model. The correlation coefficient was used to describe the relationship and direction of association between ORR and CBR with clinical variables. For the cohort of randomized trials, response rates and survival data from randomized controlled trials (RCTs) were combined in a meta-analysis using RevMan Software 5.3 (Cochrane Collaboration, Copenhagen, Denmark). In view of substantial clinical heterogeneity, estimates for ORs and HRs were pooled and weighted by generic inverse variance and computed by random effects modeling irrespective of statistical heterogeneity. Statistical heterogeneity was assessed using the Cochran’s Q and I^2^ statistics. Statistically significant heterogeneity was defined as a Cochran Q P0.10 or I^2^ > 50%. Sensitivity analysis was performed to evaluate the robustness of the results by excluding studies with high risk of bias. Statistical significance was defined as *P* < 0.05. No corrections were applied for multiple significance testing.

### Reporting summary

Further information on research design is available in the Nature Research Reporting Summary linked to this article.

## Supplementary information

SUPPLEMENTAL MATERIAL

Reporting summary

## Data Availability

The datasets generated and analyzed during the current study are available from the corresponding author on reasonable request.

## Code availability

The codes generated during the current study are available from the corresponding author on reasonable request.
