# Supplementary material for: Associations with response to Poly(ADP-ribose) Polymerase (PARP) inhibitors in patients with metastatic breast cancer
Source: NPJ Breast Cancer. 2022 Mar 31;8:43. doi: 10.1038/s41523-022-00405-1 (PMC8971397; doi:10.1038/s41523-022-00405-1)

SUPPLEMENTARY INFORMATION 1

Systematic search strategy (MEDLINE)

	**Searches**	**Results**	**Type**
1	breast cancer.af.	292411	Advanced
2	breast carcinoma.af.	25515	Advanced
3	breast malignancy.af.	710	Advanced
4	1 or 2 or 3	306449	Advanced
5	"Poly (ADP-ribose) Polymerase inhibitors".af.	4279	Advanced
6	PARP inhibitor.af.	2137	Advanced
7	"Poly (ADP-ribose) Polymerase".af.	16668	Advanced
8	PARP.af.	18535	Advanced
9	7 or 8	25186	Advanced
10	PARPi.af.	553	Advanced
11	5 or 6 or 10	5332	Advanced
12	Olaparib.af.	1677	Advanced
13	Niraparib.af.	276	Advanced
14	Rucaparib.af.	325	Advanced
15	Talazoparib.af.	234	Advanced
16	Veliparib.af.	413	Advanced
17	9 or 11 or 12 or 13 or 14 or 15 or 16	25651	Advanced
18	clinical trial.af.	735867	Advanced
19	phase 1.af.	17101	Advanced
20	phase I.af.	56379	Advanced
21	phase 2.af.	19536	Advanced
22	phase III.af.	52156	Advanced
23	phase 3.af.	15666	Advanced
24	phase II.af.	78451	Advanced
25	19 or 20	69371	Advanced
26	21 or 24	92941	Advanced
27	22 or 23	62525	Advanced
28	18 or 25 or 26 or 27	828733	Advanced
29	4 and 17 and 28	243	Advanced

SUPPLEMENTARY INFORMATION 2

Supplementary Table 1: Assessment of risk of bias of included studies

Study	Risk of bias domains					
	D1	D2	D3	D4	D5	Overall
Sandhu et al, 2013						
Balmana et al, 2014						
Fong et al, 2009						
Gelmon et al, 2011						
ICEBERG1, 2010						
Kaufman et al, 2015						
MEDIOLA, 2020						
OlympiAD, 2019						
TBCRC048, 2020						
Dent et al, 2013						
SOLACE, 2019						
Yonemori et al, 2019						
Drew et al, 2016						
Kristeleit et al, 2017						
ABRAZO, 2018						
De bono et al, 2017						

EMBRACA, 2020						
Appleman et al, 2019						
BROCADE, 2018						
BROCADE3, 2020						
Pahuja et al, 2015						
Puhalla et al, 2014						
Rodler et al, 2016						
Somlo et al, 2017						
Stoller et al, 2017						
Kummar et al, 2016						
Anampa et al, 2018						
Berlin et al, 2018						
Del Conte et al, 2014						
Gruber et al, 2019						
Hafez et al, 2020						
JAVELIN PARP Medley, 2020						
Kummar et al, 2012						
Lee et al, 2017						
Lee et al, 2016						
LUCY, 2020						
Matulonis et al, 2017						

Pothuri et al, 2020						
Shimomura et al, 2019						
Stringer-Reasor et al, 2021						
TOPACIO, 2019						
Werner et al, 2018						
Wesolowski et al, 2014						

Domains:

D1: Bias due to randomization/selection of participants

D2: Bias due to deviation from intended intervention

D3: Bias due to missing data

D4: Bias due to outcome measurement

D5: Bias due to selection of reported result

Judgement:

Red: High

Yellow: Some concern

Green: Low

SUPPLEMENTARY INFORMATION 3

Supplementary Figure 1: Funnel Plots of included RCTs

a) PFS

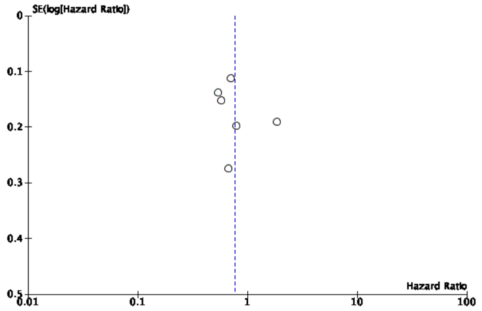

b) OS

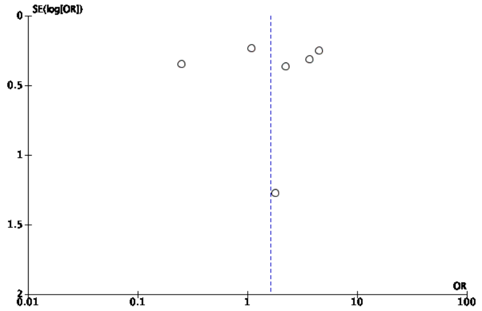

c) ORR

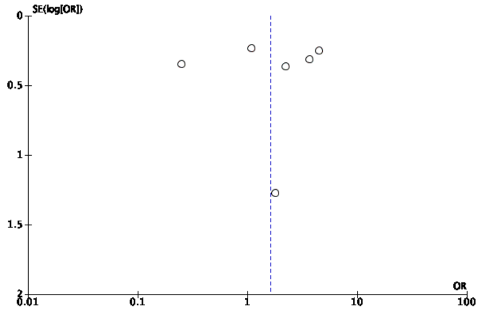

d) CBR

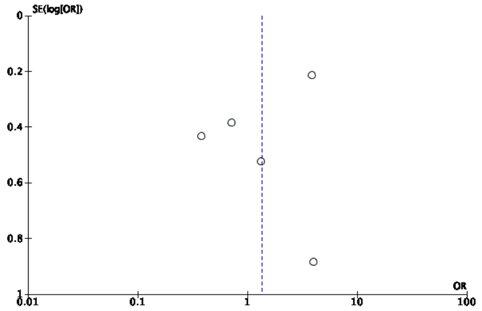

Supplement: Supplementary file 1 — SUPPLEMENTAL MATERIAL [file 41523_2022_405_MOESM1_ESM.pdf]
