# Supplementary material for: Associations with response to Poly(ADP-ribose) Polymerase (PARP) inhibitors in patients with metastatic breast cancer
Source: NPJ Breast Cancer. 2022 Mar 31;8:43. doi: 10.1038/s41523-022-00405-1 (PMC8971397; doi:10.1038/s41523-022-00405-1)
Supplement: Supplementary file 2 — Reporting summary [file 41523_2022_405_MOESM2_ESM.pdf]

nature portfolio

Corresponding author(s): Eitan Amir

Last updated by author(s): Feb 9, 2022

Reporting Summary

Nature Portfolio wishes to improve the reproducibility of the work that we publish. This form provides structure for consistency and transparency in reporting. For further information on Nature Portfolio policies, see our Editorial Policies and the Editorial Policy Checklist.

Statistics

For all statistical analyses, confirm that the following items are present in the figure legend, table legend, main text, or Methods section.

n/a	Confirmed	
☒	☐	The exact sample size (n) for each experimental group/condition, given as a discrete number and unit of measurement
☐	☒	A statement on whether measurements were taken from distinct samples or whether the same sample was measured repeatedly
☐	☒	The statistical test(s) used AND whether they are one- or two-sided<br>*Only common tests should be described solely by name; describe more complex techniques in the Methods section.*
☐	☒	A description of all covariates tested
☐	☒	A description of any assumptions or corrections, such as tests of normality and adjustment for multiple comparisons
☐	☒	A full description of the statistical parameters including central tendency (e.g. means) or other basic estimates (e.g. regression coefficient) AND variation (e.g. standard deviation) or associated estimates of uncertainty (e.g. confidence intervals)
☐	☒	For null hypothesis testing, the test statistic (e.g. F, t, r) with confidence intervals, effect sizes, degrees of freedom and P value noted<br>*Give P values as exact values whenever suitable.*
☒	☐	For Bayesian analysis, information on the choice of priors and Markov chain Monte Carlo settings
☒	☐	For hierarchical and complex designs, identification of the appropriate level for tests and full reporting of outcomes
☐	☒	Estimates of effect sizes (e.g. Cohen's d, Pearson's r), indicating how they were calculated

Our web collection on statistics for biologists contains articles on many of the points above.

Software and code

Policy information about availability of computer code

Data collection	Excel
Data analysis	SAS, RevMed

For manuscripts utilizing custom algorithms or software that are central to the research but not yet described in published literature, software must be made available to editors and reviewers. We strongly encourage code deposition in a community repository (e.g. GitHub). See the Nature Portfolio guidelines for submitting code & software for further information.

Data

Policy information about availability of data

All manuscripts must include a data availability statement. This statement should provide the following information, where applicable:

- Accession codes, unique identifiers, or web links for publicly available datasets
- A description of any restrictions on data availability
- For clinical datasets or third party data, please ensure that the statement adheres to our policy

The datasets generated during and/or analysed during the current study are available from the corresponding author on reasonable request.

Field-specific reporting

Please select the one below that is the best fit for your research. If you are not sure, read the appropriate sections before making your selection.

☒ Life sciences ☐ Behavioural & social sciences ☐ Ecological, evolutionary & environmental sciences

For a reference copy of the document with all sections, see nature.com/documents/nr-reporting-summary-flat.pdf

Life sciences study design

All studies must disclose on these points even when the disclosure is negative.

Sample size	N/A
Data exclusions	N/A
Replication	N/A
Randomization	N/A
Blinding	N/A

Reporting for specific materials, systems and methods

We require information from authors about some types of materials, experimental systems and methods used in many studies. Here, indicate whether each material, system or method listed is relevant to your study. If you are not sure if a list item applies to your research, read the appropriate section before selecting a response.

Materials & experimental systems

n/a	Involved in the study	
☒	☐	Antibodies
☒	☐	Eukaryotic cell lines
☒	☐	Palaeontology and archaeology
☒	☐	Animals and other organisms
☒	☐	Human research participants
☐	☒	Clinical data
☒	☐	Dual use research of concern

Methods

n/a	Involved in the study	
☒	☐	ChIP-seq
☒	☐	Flow cytometry
☒	☐	MRI-based neuroimaging

Clinical data

Policy information about clinical studies

All manuscripts should comply with the ICMJE guidelines for publication of clinical research and a completed CONSORT checklist must be included with all submissions.

Clinical trial registration	N/A
Study protocol	N/A
Data collection	A systematic review consistent with Preferred Reporting Items for Systematic Reviews and Meta-Analyses (PRISMA) criteria was performed(7). We searched EMBASE and MEDLINE databases from inception to March 18, 2021. A targeted search was performed to identify clinical studies that reported response rate for PARPi in patients with metastatic breast cancer. Inclusion criteria comprised adult women with inoperable locally advanced or metastatic breast cancer treated with a PARPi in the palliative setting. Studies included in the current analysis met additional predefined eligibility criteria (see Table 1). The detailed search strategy is reported in APPENDIX 1. Citations retrieved through the literature search were screened initially for inclusion based on their title and abstract. Full texts were obtained for citations that met the inclusion criteria where it was not possible to determine whether the study fulfilled inclusion criteria based on the abstract alone.<br>Data extraction was performed by a single researcher (AD). Outcome data were extracted from the primary publication and/or supplemental appendices for objective response rate (ORR), clinical benefit rate (CBR), progression-free survival (PFS), and overall survival (OS). For response rate, we collected the exact number of events and the total number of patients included in the analysis. For PFS and OS, we extracted the hazard ratio (HR) and respective 95% confidence interval (CI). If the HR was not reported explicitly, it was estimated from survival curves using the Parmar toolkit(8). Additionally, we collected the generic name of PARPi, combination drug (if any), sample size and the following study level patient characteristics: median age, number of previous lines in metastatic setting, previous platinum chemotherapy, platinum sensitivity as defined by individual trials, proportion with hormone receptor positive or triple negative disease and presence of germline BRCA1 and BRCA2 mutations.
Outcomes	The objectives of this study were to pool and to compare the activity and efficacy of PARPi for MBC in the overall population.

Outcomes

Exploratory subgroups analyses were performed to investigate if activity differed based on hormone receptor status, BRCA mutation status, prior exposure to platinum-based chemotherapy, platinum sensitivity and choice of PARPi.
